# Added Value of a New Strain Elastography Technique in Conventional Ultrasound for the Diagnosis of Breast Masses: A Prospective Multicenter Study

**DOI:** 10.3389/fonc.2021.779612

**Published:** 2021-11-09

**Authors:** Qi Wei, Yu-Jing Yan, Ge-Ge Wu, Xi-Rong Ye, Fan Jiang, Jie Liu, Gang Wang, Yi Wang, Yu Wang, Zhi-Ping Pan, Jin-Hua Hu, Juan Song, Christoph F. Dietrich, Xin-Wu Cui

**Affiliations:** ^1^ Sino-German Tongji-Caritas Research Center of Ultrasound in Medicine, Department of Medical Ultrasound, Tongji Hospital, Tongji Medical College, Huazhong University of Science and Technology, Wuhan, China; ^2^ Department of Medical Ultrasound, The Central Hospital of EDong Healthcare, Huangshi, China; ^3^ Department of Medical Ultrasound, The Second Hospital of Anhui Medical University, Hefei, China; ^4^ Department of Medical Ultrasound, Yichang General Hospital, Renmin Hospital of Three Gorges University, Yichang, China; ^5^ Department of Medical Ultrasound, Taizhou Hospital of Zhejiang Province, Linhai, China; ^6^ Department of Medical Ultrasound, Macheng People’s Hospital, Macheng, China; ^7^ Department of Medical Ultrasound, Xiangyang No. 1 People’s Hospital, Affiliated Hospital of Hubei University of Medicine, Xiangyang, China; ^8^ Department of Medical Ultrasound, Yixing Traditional Chinese Medicine Hospital, Yixing, China; ^9^ Department of Medical Ultrasound, Anqing First People’s Hospital of Anhui Medical University, Anqing, China; ^10^ Department of Internal Medicine, Hirslanden Clinic, Bern, Switzerland

**Keywords:** strain elastography, elasticity score, strain ratio, ultrasound, breast masses

## Abstract

**Objective:**

This study aimed to explore the value of elasticity score (ES) and strain ratio (SR) combined with conventional ultrasound in distinguishing benign and malignant breast masses and reducing biopsy of BI-RADS (Breast Imaging Reporting and Data System) 4a lesions.

**Methods:**

This prospective, multicenter study included 910 patients from nine different hospitals. The acquisition and analysis of conventional ultrasound and strain elastography (SE) were obtained by radiologists with more than 5 years of experience in breast ultrasound imaging. The diagnostic sensitivity, specificity, positive predictive value (PPV), negative predictive value (NPV), and area under curve (AUC) of conventional ultrasound alone and combined tests with ES and/or SR were calculated and compared.

**Results:**

The optimal cutoff value of SR for differentiating benign from malignant masses was 2.27, with a sensitivity of 60.2% and a specificity of 84.8%. When combined with ES and SR, the AUC of the new BI-RADS classification increased from 0.733 to 0.824 (*p* < 0.001); the specificity increased from 48.1% to 68.5% (*p* < 0.001) without a decrease in the sensitivity (98.5% *vs*. 96.4%, *p* = 0.065); and the PPV increased from 52.2% to 63.7% (*p* < 0.001) without a loss in the NPV (98.2% *vs*. 97.1%, *p* = 0.327). All three combinations of conventional ultrasound, ES, and SR could reduce the biopsy rate of category 4a lesions without reducing the malignant rate of biopsy (from 100% to 68.3%, 34.9%, and 50.4%, respectively, all *p* < 0.001).

**Conclusions:**

SE can be used as a useful and non-invasive additional method to improve the diagnostic performance of conventional ultrasound by increasing AUC and specificity and reducing the unnecessary biopsy of BI-RADS 4a lesions.

## Introduction

The morbidity of breast cancer is the highest in the world, and the mortality ranks fifth among all cancers but first in female cancers (1). Early detection and timely diagnosis of breast cancer are closely related to the prognosis of patients. Ultrasound is widely used in the examination of patients with breast abnormalities. However, the lack of specificity of B-mode ultrasound in the diagnosis of breast masses leads to unnecessary biopsy (2), which leads to negative effects such as pain, anxiety, and complications (3).

Strain elastography (SE) is easily performed and provides elastic images with a high spatial resolution by evaluating tissue deformation (4). In general, malignant breast tissue is harder than normal breast tissue and produces less strain (5). Differentiating benign and malignant breast masses and upgrading or downgrading the Breast Imaging Reporting and Data System (BI-RADS) classification to avoid unnecessary biopsy are clinical indications for elastography according to the WFUMB guidelines and recommendations for clinical use of ultrasound elastography to breast (6). Ultrasound elastography technique may improve the specificity of B-mode ultrasound in the differential diagnosis of breast masses by measuring tissue stiffness (2, 7), even for breast masses smaller than 1 cm in diameter (8). Elasticity assessment has been incorporated into the fifth edition of BI-RADS lexicon to further describe the characteristics of breast masses (9). The combination of conventional ultrasound and SE can reduce unnecessary biopsy of breast masses by down-staging the BI-RADS classification (10). SE was strongly recommended as a supplementary diagnostic tool for conventional ultrasound by the latest EFSUMB (European Federation of Societies for Ultrasound in Medicine and Biology) guidelines and recommendations for the clinical practice of elastography for non-hepatic applications released in 2018 (11). Three diagnostic methods of SE including elasticity score (ES), strain ratio (SR), and strain size ratio (EI/B ratio) were mainly used to classify breast lesions in clinic (6).

Most previous studies explored the value of SE in breast masses using Hitachi ultrasound equipment (5, 12, 13). However, the SE in different brands of ultrasound systems has different reference standards for clinical use. Recently, a new SE technique, with the function of measuring ES and SR, has been equipped in Samsung ultrasound systems. At present, only one single study has explored the diagnostic performance of SE of this system in differentiating benign and malignant breast masses (14). More studies are needed to explore the added value of ES and SR in the differential diagnosis of breast masses.

The prospective multicenter study aimed to determine the cutoff value of SR and to explore the value of ES and SR in combination with conventional ultrasound in distinguishing benign and malignant breast masses and reducing biopsy of BI-RADS 4a lesions.

## Materials and Methods

This prospective multicenter study enrolled patients from nine institutions in different regions of China between April 2019 and November 2020. It was approved by the ethics committee of Tongji Medical College of Huazhong University of Science and Technology and registered in ClinicalTrials.gov (NCT 03887598). The informed consent of all participants was obtained in this study.

### Participants

The inclusion criteria of participants were as follows: (i) patients had definite pathological results after ultrasound examination, and (ii) patients were at least 18 years old. The exclusion criteria were as follows: (i) patients who received radiotherapy or chemotherapy before the examination; (ii) patients who did not have reliable SE images or SR analyses; and (iii) patients who were lactating or pregnant.

### Image Acquisition: B-Mode Ultrasound and SE

Conventional ultrasound imaging and SE technique were performed with the Samsung RS80A ultrasound system (Samsung Madison Co., Ltd., Seoul, South Korea) in all patients. The acquisition of ultrasound images and the analysis of SE images were performed by nine radiologists with more than 5 years of experience in breast ultrasound imaging. The standard data acquisition process was established, and all operators received rigorous training before the enrollment of patients. The study was conducted only after five qualified test cases were uploaded from every single center and checked by the principle investigator.

**Figure 1:**
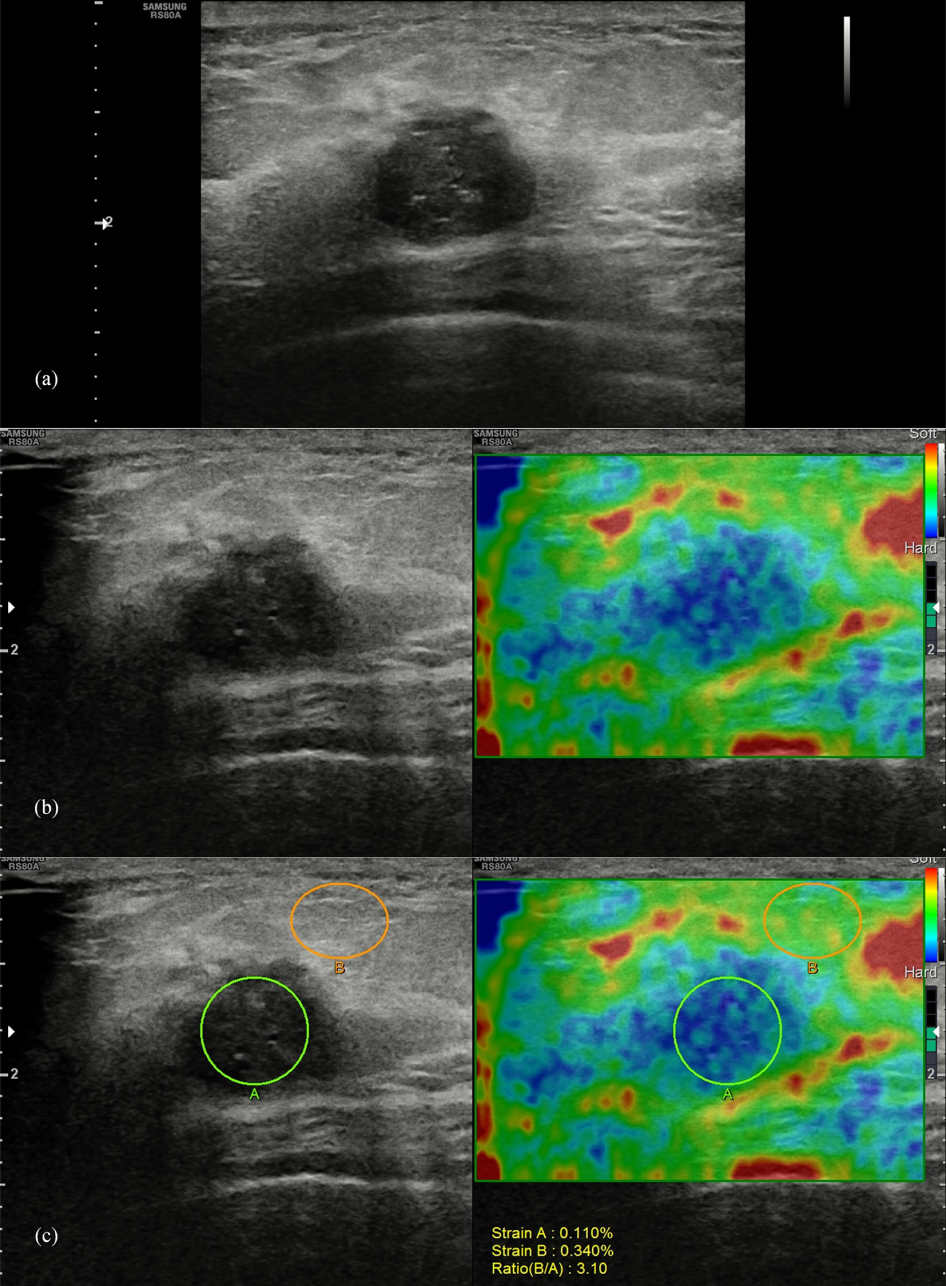
Pathologically confirmed breast invasive ductal carcinoma in a 44-year-old female patient. Ultrasound images of the long-axis section of the breast mass were evaluated as BI-RADS 4c **(A)**, with an elasticity score of 4 **(B)**, and a strain ratio of 3.1 **(C)**.

Breast B-mode ultrasound was performed in the supine position in all patients using a 3–12 MHz linear transducer. B-mode videos of the lesions were documented in both the long axis and short axis. SE imaging was performed using the same 3–12 MHz linear transducer based on WFUMB guidelines (6). The SE images were obtained by manually applying slight vibration with the probe perpendicular to the skin under the guidance of the quality indicator. After the elastic image was stabilized, SR and ES were acquired on a representative static image by the same operator. Strain A was obtained by placing the ROI in the target mass, and strain B was obtained by placing the ROI in the subcutaneous fibroglandular tissue at the same depth as the mass (**Figure 1**). The SR (the ratio of strain B to strain A) calculated by the system was recorded. SE videos of the lesions were documented in both the long axis and short axis.

### Image Analysis

Conventional ultrasound features of breast masses were analyzed by two experienced radiologists (more than 5 years of experience in breast ultrasound imaging) who were blinded to the pathological results according to BI-RADS classification (12) and finally evaluated as category 2, 3, 4a, 4b, 4c, and 5. The final assessments would be given after a discussion of two radiologists when there was a disagreement. Category 4a was considered as the cutoff value: benign, category 2 or 3; malignant, category 4a, 4b, 4c, or 5.

The elastic scoring criteria of breast masses are shown in **Table 1** (8). Scores 1 to 3 were considered benign, while scores 4 and 5 were considered malignant. The optimum cutoff value of SR was determined by receiver operating characteristic (ROC) analysis. A breast mass was considered malignant when the SR value was higher than the cutoff value. Otherwise, it was considered benign.

**Table 1:** The elastic scoring criteria of breast masses.

Elasticity score	Description
Score 1	Homogeneous green within the mass
Score 2	Most of the area is light green, with some blue around and/or in the center of the mass
Score 3	Half of the area is blue and half is green in the mass
Score 4	Homogeneously blue with or without a little green within the mass
Score 5	Homogeneously blue with or without a little green throughout the entire mass and its surrounding area

### Combination Criteria of B-Mode Ultrasound and ES and/or SR

The combined analysis of B-mode ultrasound, ES, and SR of all images was based on the long-axis section of the breast mass. The BI-RADS classification of the breast mass was reassessed when combined with the ES and SR. Only BI-RADS categories 3 and 4a were upgraded or downgraded in this study. When conventional ultrasound was combined with ES or SR, BI-RADS category 3 was upgraded to category 4a if the result was malignant; BI-RADS category 4a would be downgraded to category 3 if benign was recommended. When conventional ultrasound was combined with ES and SR, category 3 was upgraded to category 4a if both ES and SR results are malignant; category 4a would be downgraded to category 3 if both were recommended as benign; otherwise, the BI-RADS classification of the mass would be unchanged.

### Statistical Analysis

The histopathological results were considered the reference standard for this study. The diagnostic sensitivity, specificity, positive predictive value (PPV), negative predictive value (NPV), area under the curve (AUC) value, and positive and negative diagnostic likelihood ratios (LR+, LR-) of ES and SR on two different sections (long axis and short axis) were calculated and compared. The diagnostic value of the combination of conventional ultrasound and ES and/or SR in differentiating benign and malignant breast masses and reducing biopsy of BI-RADS 4a lesions were analyzed and compared: conventional ultrasound and ES, conventional ultrasound and SR, and conventional ultrasound and ES and SR.

Quantitative data such as patient age and tumor size were expressed as means and standard deviations, and compared using *t* test or Mann–Whitney *U* test. The chi-square test and Fisher’s test were used to compare categorical variables. The comparison between AUC values was performed by the DeLong method (15). The SPSS (version 22, IBM Corp.) and MedCalc software (V.19.0.7, MedCalc Software, Ostend, Belgium) were used for all statistical analyses. *p*-values less than 0.05 were assumed to be statistically significant.

## Results

### Clinical Characteristics

**Figure 2:**
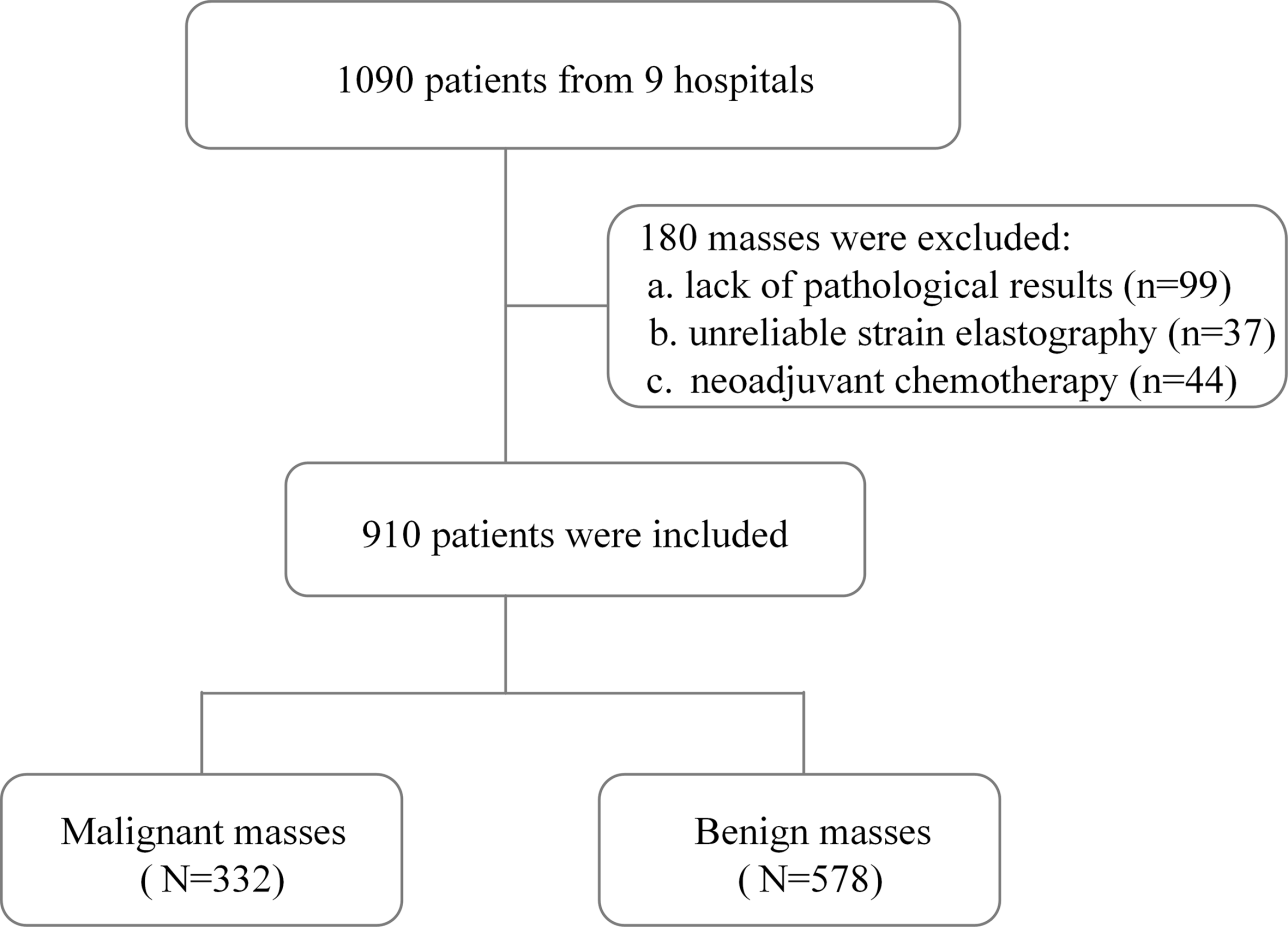
Flowchart of patient selection in the study.

A total of 910 patients (mean age, 45.3 ± 10.9 years) were finally included in this study after the exclusion criteria were performed (**Figure 2**). Invasive ductal carcinoma was the most common of 332 (36.5%) malignant breast masses, accounting for 83.4% (277/332). Among 578 (63.5%) cases of benign breast masses, proliferative disease (61.2%, 354/578) and fibroadenoma (31.0%, 179/578) were the main ones. The characteristics of patients and masses are summarized in **Table 2**. Patients with benign breast masses are significantly younger than those with malignant masses (42.3 ± 9.9 *vs*. 50.4 ± 10.5, *p* < 0.001). The diameter (22.5 ± 10.4 *vs*. 14.6 ± 7.3, *p* < 0.001) and SR (2.8 ± 1.6 *vs*. 1.6 ± 0.8, *p* < 0.001) of the breast mass with histopathological findings of malignancy were significantly higher than those of the benign mass. In addition, there were significant statistical differences in the distribution of malignant and benign breast masses in the ES and BI-RADS classification (all *p* < 0.001).

**Table 2:** The characteristics of patients and breast masses.

Characteristics	All masses	Malignant	Benign	*p*
Mean age, years	45.3 ± 10.9	50.4 ± 10.5	42.3 ± 9.9	<0.001
Mean tumor size, mm*	17.5 ± 9.3	22.5 ± 10.4	14.6 ± 7.3	<0.001
Size <20 mm	617	148	469	
Size ≥20 mm	293	184	109	
Strain ratio*	2.1 ± 1.3	2.8 ± 1.6	1.6 ± 0.8	<0.001
Elasticity score (ES) *				<0.001
ES 1	19	0	19	
ES 2	343	28	315	
ES 3	66	8	58	
ES 4	459	273	186	
ES 5	23	23	0	
BI-RADS classification*				<0.001
Category 3	283	5	278	
Category 4a	252	29	223	
Category 4b	89	37	52	
Category 4c	99	82	17	
Category 5	187	179	8	

*Data obtained based on the long-axis section of the mass.

### SR in BI-RADS Classification

**Figure 3:**
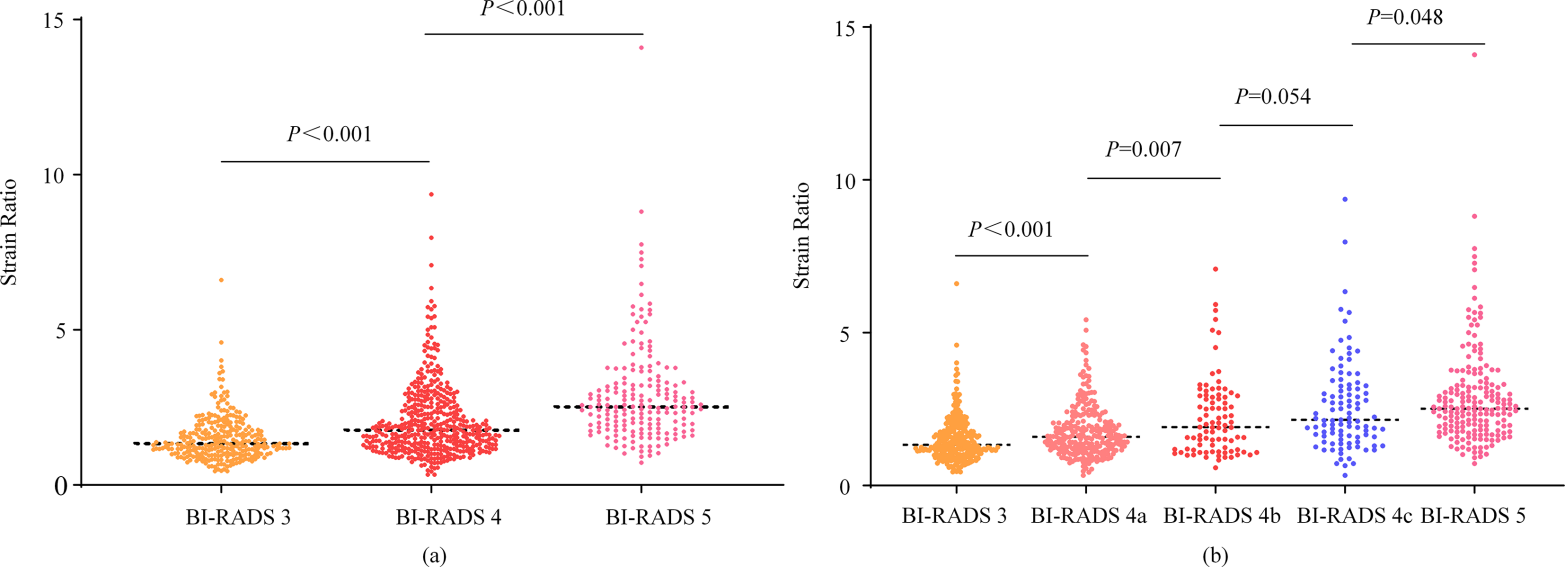
Scatter plots of strain ratios in different BI-RADS classifications. The Mann-Whitney U test was used to compare the difference in strain ratio between different BI-RADS classifications.

SR values of different BI-RADS categories are shown in **Table 3**. For masses classified as BI-RADS category 5, the SR value was significantly higher than that of category 4 (median value, 2.520 *vs*. 1.770, *p* < 0.001), and the SR value for category 4 masses was significantly higher than that of category 3 (median value, 1.770 vs. 1.330, *p* < 0.001, **Table 3** and **Figure 3A**). The median value of SR increased with the increase of BI-RADS classification. When BI-RADS 4 were sub-categorized as 4a, 4b, and 4c, the SR value of category 5 was higher than category 4c (median value, 2.520 *vs*. 2.150, *p* = 0.048), category 4b was higher than category 4a (median value, 1.910 *vs*. 1.595, *p* = 0.007), and category 4a was higher than category 3 (median value, 1.595 vs. 1.330, *p* < 0.001). However, there was no statistical difference in the SR value between category 4b and 4c (median value, 2.150 *vs*. 1.910, *p* = 0.054, **Table 3** and **Figure 3B**).

**Table 3:** The strain ratio in different BI-RADS classifications.

Strain Ratio	BI-RADS 3	BI-RADS 4				BI-RADS 5
		All	BI-RADS 4a	BI-RADS 4b	BI-RADS 4c	
Median value	1.330	1.770	1.595	1.910	2.150	2.520
Interquartile range	(1.020, 1.790)	(1.250, 2.568)	(1.210, 2.108)	(1.185, 2.915)	(1.600, 3.250)	(1.860, 3.280)

### Diagnostic Performance of ES and SR

The optimal cutoff values of SR in the long-axis and short-axis sections were determined by the Youden index. In the long-axis section of the breast mass, 2.27 was the optimal cutoff value of SR, with a sensitivity of 60.2% and a specificity of 84.8%. In the short-axis section, 2.12 was the optimal cutoff value of SR, with a sensitivity of 63.6% and a specificity of 82.5% (**Table 4**). The AUC of SR on the long-axis and short-axis sections was 0.787 and 0.786, respectively. The AUC of ES on the long-axis and short-axis sections was 0.829 and 0.817, respectively. There was no statistical difference in the diagnostic performance of SR in different sections of breast masses, with the *p* values all greater than 0.05. Similarly, the diagnostic performance of ES was not affected by different planes of breast masses (all *p* > 0.05, **Table 4**).

**Table 4:** Comparison of the diagnostic performance of elasticity score and strain ratio.

Parameter	SR			ES			Long axis	Short axis
	Long axis	Short axis	*p*	Long axis	Short axis	*p*	*p* (SR *vs*. ES)	*p* (SR *vs*. ES)
Cutoffs	2.27	2.12						
AUC	0.787 (0.759, 0.814)	0.786 (0.758, 0.812)	0.928	0.829 (0.803, 0.853)	0.817 (0.790, 0.841)	0.229	0.003	0.028
Sensitivity, %	60.2 (54.8, 65.5)	63.6 (58.1, 68.7)	0.278	89.2 (85.3, 92.3)	88.9 (85.0, 92.0)	>0.999	<0.001	<0.001
Specificity, %	84.8 (81.6, 87.6)	82.5 (79.2, 85.5)	0.223	67.8 (63.8, 71.6)	64.0 (60.0, 67.9)	0.053	<0.001	<0.001
PPV, %	69.4 (64.8, 73.7)	67.6 (63.2, 71.7)	0.632	61.4 (58.4, 64.3)	58.6 (55.8, 61.4)	0.376	0.024	0.010
NPV, %	78.8 (76.4, 81.0)	79.8 (77.3, 82.0)	0.671	91.6 (88.8, 93.7)	90.9 (88.0, 93.2)	0.728	<0.001	<0.001
LR+	4.0 (3.2, 4.9)	3.6 (3.0, 4.4)		2.8 (2.4, 3.1)	2.5 (2.2, 2.8)			
LR-	0.5 (0.4, 0.5)	0.4 (0.4, 0.5)		0.2 (0.1, 0.2)	0.2 (0.1, 0.2)			

SR, strain ratio; ES, elasticity score; AUC, the area under curve; PPV, positive predictive value; NPV, predictive value.

LR+, positive diagnostic likelihood ratios; LR-, negative diagnostic likelihood ratios.

95% confidence interval in parentheses.

**Figure 4:**
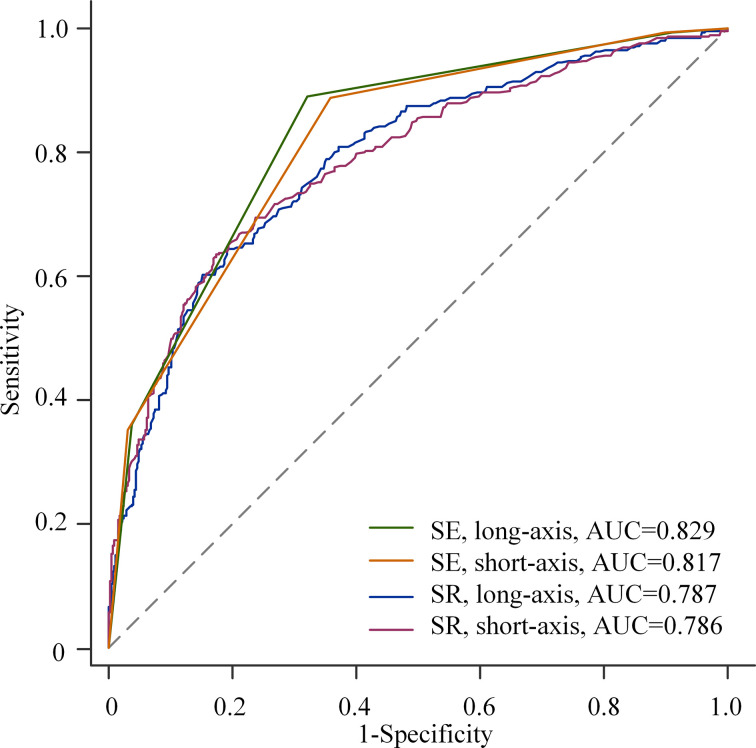
Receiver operating characteristic (ROC) curves for elasticity score (ES) and strain ratio (SR) in different planes.

Compared with SR, ES showed higher AUC (0.829 *vs*. 0.787, *p* = 0.003; 0.817 *vs*. 0.786, *p* = 0.028), sensitivity (89.2% *vs*. 60.2%, *p* < 0.001; 88.9% *vs*. 63.6%, *p* < 0.001), and NPV (91.6% *vs*. 78.8%, *p* < 0.001; 90.9% vs. 79.8%, *p* < 0.001), and lower specificity (67.8% *vs*. 84.8%, *p* < 0.001; 64.0% *vs*. 82.5%, *p* < 0.001) and PPV (61.4% *vs*. 69.4%, *p* = 0.024; 58.6% *vs*. 67.6%, *p* = 0.010) in different planes (**Table 4**). The comparison of ROC curves between ES and SR is shown in **Figure 4**.

### Diagnostic Value of BI-RADS Combined ES and SR

When combined with ES and SR, the diagnostic performance of the re-assessed BI-RADS classification was as follows: the AUC increased from 0.733 to 0.824 (*p* < 0.001); the specificity increased from 48.1% to 68.5% (*p* < 0.001) without a decrease in the sensitivity (98.5% *vs*. 96.4%, *p* = 0.065), and the PPV increased from 52.2% to 63.7% (*p* < 0.001) without a loss in the NPV (98.2% *vs*. 97.1%, *p* = 0.327, **Table 5**).

**Table 5:** Diagnostic value of BI-RADS and combined tests with strain ratio and/or elasticity score.

Parameter	BI-RADS	B+ES		B+SR		B+ES+SR	
		Combined	p*	Combined	p*	Combined	p*
AUC	0.733 (0.703, 0.761)	0.783 (0.755, 0.810)	<0.001	0.846 (0.821, 0.869)	<0.001	0.824 (0.798, 0.849)	<0.001
Sensitivity, %	98.5 (96.5, 99.5)	96.1 (93.4, 97.9)	0.039	94.9 (91.9, 97.0)	0.004	96.4 (93.8, 98.1)	0.065
Specificity, %	48.1 (44.0, 52.3)	60.6 (56.4, 64.6)	<0.001	74.4 (70.6, 77.9)	<0.001	68.5 (64.6, 72.3)	<0.001
PPV, %	52.2 (50.2, 54.1)	58.3 (55.8, 60.8)	0.034	68.0 (64.9, 71.0)	<0.001	63.7 (60.9, 66.5)	<0.001
NPV, %	98.2 (95.9, 99.3)	96.4 (94.0, 97.9)	0.164	96.2 (94.1, 97.6)	0.117	97.1 (95.0, 98.3)	0.327
LR+	1.9 (1.8, 2.1)	2.4 (2.2, 2.7)	NA	3.7 (3.2, 4.3)	NA	3.1 (2.7, 3.5)	NA
LR-	0.03 (0.01, 0.08)	0.07 (0.04, 0.1)	NA	0.07 (0.04, 0.1)	NA	0.05 (0.03, 0.09)	NA

B, BI-RADS; ES, elasticity score; SR, strain ratio; AUC, the area under curve.

PPV, positive predictive value; NPV, negative predictive value.

LR+, positive diagnostic likelihood ratios; LR-, negative diagnostic likelihood ratios; NA, not applicable.

*Compared with BI-RADS classification alone. 95% confidence interval in parentheses.

**Figure 5:**
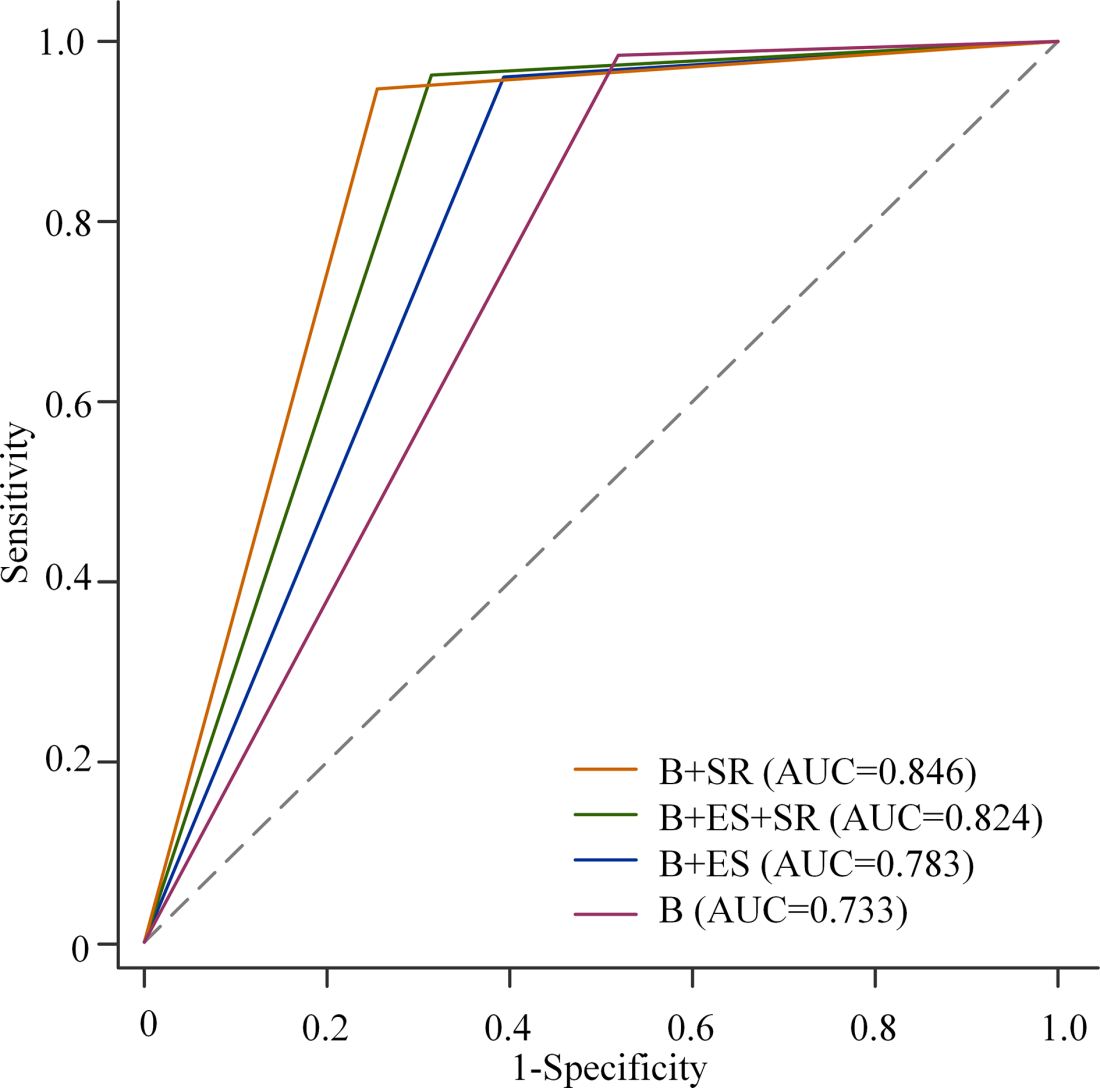
Receiver operating characteristic (ROC) curves for B-mode ultrasound (B) alone, B-mode ultrasound combined with elasticity score (ES), and/or strain ratio (SR). Among the three combinations, the AUC of BI-RADS classification combined with SR was the highest, and that of BI-RADS classification combined with SE was the lowest (0.846 *vs*. 0.824, *p* = 0.001; 0.846 vs. 0.783, *p* < 0.001; 0.824 *vs*. 0.783, *p* < 0.001).

The AUC, specificity, and PPV were higher than those of BI-RADS classification alone by the addition of ES or SR to the BI-RADS classification (all *p* < 0.05). However, the sensitivity decreased from 98.5% to 96.1% (BI-RADS combined with ES, *p* = 0.039) and 94.9% (BI-RADS combined with SR, *p* = 0.004), respectively. There was no statistically significant difference in NPV, with all *p* > 0.05 (**Table 5**). The comparison of the AUC of the three combination methods is shown in **Figure 5**.

### The Value of ES and SR in Reducing Unnecessary Biopsy

The value of ES and SR in reducing biopsy of BI-RADS 4a lesions is summarized in **Table 6**. When BI-RADS was combined with ES, biopsy was avoided in 80 masses, and the biopsy rate decreased from 100% to 68.3% (*p* < 0.001). When combined with SR, 164 masses avoided biopsy, with a lower biopsy rate (34.9% vs. 100%, *p* < 0.001). When combined with both ES and SR, biopsy of 125 masses was avoided and the biopsy rate decreased to 50.4% (*p* < 0.001). There was no statistical difference in the malignant rate of biopsy regardless of the combination of BI-RADS and SE (all *p* > 0.05).

**Table 6:** The value of ES and SR in reducing biopsy of BI-RADS 4a lesions.

Parameter	BI-RADS	B+ES		B+SR		B+ES+SR	
		Combined	p*	Combined	p*	Combined	p*
Number of 4a lesions	252	172		88		127	
Number of 3 to 4a		80		29		23	
Number of 4a to 3		160		193		148	
Masses avoid biopsy	0	80		164		125	
Biopsy rate, %	100 (252/252)	68.3 (172/252)	<0.001	34.9 (88/252)	<0.001	50.4 (127/252)	<0.001
Malignant rate of biopsy, %	11.5 (29/252)	12.2 (21/172)	0.826	19.3 (17/88)	0.065	17.3 (22/127)	0.117

B, BI-RADS; ES, elasticity score; SR, strain ratio.

*Compared with BI-RADS classification alone.

## Discussion

This prospective multicenter study explored the auxiliary value of two SE diagnostic methods, ES and SR, in the assessment of B-mode ultrasound breast lesions. Our results indicate that ES has a higher AUC, sensitivity, and NPV, but lower specificity and PPV in differentiating benign from malignant breast masses compared with SR. The AUC, specificity, and PPV of BI-RADS combined with ES and SR were higher than those of BI-RADS alone, without the loss of sensitivity and NPV. In addition, BI-RADS combined with ES or/and SR can significantly reduce the biopsy rate of BI-RADS 4a lesions without affecting the malignant rate of biopsy.

Recently, a new SE technique equipped in Samsung ultrasound system has been more and more widely used, and a multicenter study to explore how to make better use of its strain technique is necessary. Studies have shown that the ES and SR of SE techniques show good diagnostic accuracy in distinguishing benign from malignant breast masses (16, 17). These are consistent with our result that both ES and SR have statistical differences in the differentiation between benign and malignant breast masses. However, 3.5 (10), 4.2 (16), 2.3 (17), and 4.5 (18) were used as cutoff values of SR to distinguish benign and malignant breast masses. The difference in the cutoff value of SR may be caused by the difference in strain calculation methods of various equipment vendors (17). In addition, the measurement of SR is greatly affected by the initial shear modulus and elastic nonlinearity of the lesion, as well as the pre-compression during image acquisition (19). In the previous single-center study, the cutoff value of SR was 1.765, and the sensitivity and specificity were 76% and 75%, respectively. In our multicenter study, 2.27 was the optimal cutoff value of SR, with a sensitivity of 60.2% and a specificity of 84.8%. The data for this study come from nine different hospitals, and the cutoff value of SR may be more objective.

The influence of different planes of breast masses on elastography imaging was discussed in this study. The results showed that there were no statistical differences in the diagnostic performance of both ES and SR in the long-axis and short-axis sections, which reflected the stability of SE. With respect to the diagnostic performance of ES and SR in the diagnosis of breast lesions, our study showed that ES was superior to SR, with a higher AUC (0.829 *vs*. 0.787, *p* < 0.001). A previous study showed that ES was the most useful in the identification of benign and malignant breast masses among the four diagnostic methods (ES, SR, distance ratio, and area ratio) of SE (20), which was consistent with our result. However, some studies showed that there was no significant statistical difference in the diagnostic value of ES and SR in distinguishing benign and malignant breast masses (10, 16). The operator dependence of elastography may be one of the reasons.

Elastography was considered to be helpful to improve the specificity of conventional ultrasound (11). In this study, the combination of BI-RADS and SE was in the following three forms: BI-RADS combined with ES, BI-RADS combined with SR, and BI-RADS combined with both ES and SR. Our results indicated that the combination of ES and/or SR could significantly improve the AUC and specificity of BI-RADS, which was consistent with the guidelines. Therefore, SE can be used as a useful additional method for a conventional ultrasound. Among the three combinations, the AUC of BI-RADS classification combined with SR was the highest, followed by that of BI-RADS classification combined with both ES and SR, and that of BI-RADS classification combined with SE was the lowest (0.846 *vs*. 0.824, *p* = 0.001; 0.846 *vs*. 0.783, *p* < 0.001; 0.824 *vs*. 0.783, *p* < 0.001). However, the combination of BI-RADS classification and ES or SR decreased the sensitivity compared to the BI-RADS evaluation alone (98.5% *vs*. 96.1%, *p* = 0.039; 98.5% *vs*. 94.9%, *p* = 0.004). When combined with both ES and SR, the sensitivity of the BI-RADS classification decreased from 98.5% to 96.4% with no statistical difference (*p* = 0.065). Therefore, BI-RADS classification combined with both ES and SR performed best to improve specificity without the loss of sensitivity.

The study showed that SR can be used as a valuable method for the evaluation of breast lesions in categories 3 and 4a, but not in categories 4b and 4c (21). Similarly, our results showed that the SR value for category 4a lesions was significantly higher than that for category 3 lesions, but there was no significant difference between category 4b and 4c lesions. For breast lesions that were highly suspected for malignancy by conventional ultrasound, the stiffness of the tissue had little effect on the patient’s clinical decision-making (21). Elastography may play a role in improving the selection of biopsy for patients with low suspicion lesions (20). BI-RADS category 3 or 4a lesions were upgraded or downgraded based on the results of ES and SR. This multicenter study showed that all three combined methods could reduce the biopsy rate of category 4a lesions without reducing the malignant rate of biopsy, and BI-RADS classification combined with SR was found to be the most useful. Therefore, elastography imaging can be used as a non-invasive auxiliary method to reduce unnecessary biopsy of BI-RADS 4a lesions, thus avoiding negative emotions of patients and complications of tissue biopsy.

The main limitation of our study was the uneven distribution of the patient population and histopathological results. In addition, the repeatability of the breast SE technique with the Samsung ultrasound system was not explored in this study. The repeatability of elastography is mainly manifested in the variability of data acquisition and interpretation (22). Our prospective studies followed very strict procedural protocols to minimize differences among radiologists in data acquisition and interpretation. Lastly, some patients were unable to be included in this study because they did not get a reliable SE assessment. On the one hand, breast masses do not meet the requirements of elastic quality indicators. On the other hand, the breast mass is too large and occupies the whole elastic frame, so it is impossible to evaluate the elasticity.

In summary, the optimal cutoff value of SR for differentiating benign from malignant masses was 2.27, with a sensitivity of 60.2% and a specificity of 84.8%. In addition, SE can be used as a useful and non-invasive additional method to improve the diagnostic performance of conventional ultrasound by increasing AUC and specificity and reducing unnecessary biopsy of BI-RADS 4a lesions.

## Data Availability Statement

The raw data supporting the conclusions of this article will be made available from the corresponding author upon reasonable request.

## Ethics Statement

The studies involving human participants were reviewed and approved by the Institutional Review Board of Tongji Medical College of Huazhong University of Science & Technology. The patients/participants provided their written informed consent to participate in this study.

## Author Contributions

Conception and design of the study: X-WC and CD. Acquisition of data: QW, Y-JY, X-RY, FJ, JL, GW, YiW, YuW, Z-PP, and J-HH. Analysis and interpretation of data: QW, G-GW, JS, and X-WC. Drafting the article: QW, Y-JY, and X-WC. Revising it critically for important intellectual content: all authors. All authors contributed to the article and approved the submitted version.

## Funding

This research was supported by the National Natural Science Foundation of China (Grant No. 82071953), Key R&D Projects of Science and Technology of Hubei Province in 2020 (Grant No. 2020BCB022), and Tongji Hospital (HUST) Foundation for Excellent Young Scientist (Grant No. 2020YQ01).

## Conflict of Interest

Author CD was employed by company Hirslanden Clinic.

The remaining authors declare that the research was conducted in the absence of any commercial or financial relationships that could be construed as a potential conflict of interest.

## Publisher’s Note

All claims expressed in this article are solely those of the authors and do not necessarily represent those of their affiliated organizations, or those of the publisher, the editors and the reviewers. Any product that may be evaluated in this article, or claim that may be made by its manufacturer, is not guaranteed or endorsed by the publisher.
